# Effects of Active and Passive Hearing Protection Devices on Sound Source Localization, Speech Recognition, and Tone Detection

**DOI:** 10.1371/journal.pone.0136568

**Published:** 2015-08-27

**Authors:** Andrew D. Brown, Brianne T. Beemer, Nathaniel T. Greene, Theodore Argo, G. Douglas Meegan, Daniel J. Tollin

**Affiliations:** 1 Department of Physiology & Biophysics, University of Colorado School of Medicine, Aurora, Colorado, United States of America; 2 Department of Speech, Language, and Hearing Sciences, University of Colorado-Boulder, Boulder, Colorado, United States of America; 3 Applied Research Associates, Inc., Littleton, Colorado, United States of America; UNLV, UNITED STATES

## Abstract

Hearing protection devices (HPDs) such as earplugs offer to mitigate noise exposure and reduce the incidence of hearing loss among persons frequently exposed to intense sound. However, distortions of spatial acoustic information and reduced audibility of low-intensity sounds caused by many existing HPDs can make their use untenable in high-risk (e.g., military or law enforcement) environments where auditory situational awareness is imperative. Here we assessed (1) sound source localization accuracy using a head-turning paradigm, (2) speech-in-noise recognition using a modified version of the QuickSIN test, and (3) tone detection thresholds using a two-alternative forced-choice task. Subjects were 10 young normal-hearing males. Four different HPDs were tested (two active, two passive), including two new and previously untested devices. Relative to unoccluded (control) performance, all tested HPDs significantly degraded performance across tasks, although one active HPD slightly improved high-frequency tone detection thresholds and did not degrade speech recognition. Behavioral data were examined with respect to head-related transfer functions measured using a binaural manikin with and without tested HPDs in place. Data reinforce previous reports that HPDs significantly compromise a variety of auditory perceptual facilities, particularly sound localization due to distortions of high-frequency spectral cues that are important for the avoidance of front-back confusions.

## Introduction

Hearing is important for communication and situational awareness. Hearing *impairment* can thus cause disability and reduce safety in a variety of settings [1,2]. Hearing impairment is particularly detrimental in military or law enforcement settings, potentiating reduced survivability and fitness for duty [2]. Unfortunately, due to frequent exposures to high-intensity sound, the prevalence of hearing impairment among military personnel is extraordinarily high—roughly 30%, three times the prevalence in the general population [3,4]. Although hearing protection devices (HPDs) such as earplugs and earmuffs offer to mitigate noise exposure and risk of hearing loss, HPDs can themselves reduce audibility and distort acoustic information, negatively impacting situational awareness and communication [5–9]. Many devices are also uncomfortable to wear for extended periods of time. Thus, while HPDs are widely distributed and available in military and industrial settings, they are often unused, or are used irregularly [10,11]. It is therefore desirable to develop HPDs that (1) preserve the audibility of low- to moderate-intensity sounds while attenuating high-intensity sounds, (2) preserve acoustic information that facilitates auditory situational awareness, and (3) are comfortable to wear.

Conventional, level-independent HPDs such as high-density foam earplugs attenuate sound in a roughly linear manner. Thus, while dangerous high-intensity sounds are attenuated to safer levels, important low-intensity sounds may be attenuated to inaudible levels [12]. Many currently available more technologically advanced HPDs are *non*linear (“level-dependent”), attenuating high-intensity sounds relatively more than low-intensity sounds. Such nonlinearity may arise via passive acoustic features of the device (e.g., narrow-diameter vents, which have particle velocity-dependent impedance, and thus limit the passage of high-intensity sound) or from active processing (e.g., nonlinear amplification) via onboard electronics [12].

While level-dependent HPDs are thus generally improved over level-independent HPDs, a variety of persistent deleterious auditory perceptual effects of HPDs have been noted. For example, most devices still cause some attenuation, and reduce the detectability of low-intensity (or distant) sources [7]. Relatedly, HPDs can also significantly degrade speech intelligibility for people with concomitant hearing loss [13], although normal-hearing individuals may experience improvements in speech intelligibility in noisy environments due to the broadband attenuation of background noise provided by HPDs [13–15].

More seriously, HPDs distort the spatial acoustic cues that the auditory system normally exploits for sound source localization and segregation. These include interaural time and level differences (ITD and ILD) and spectral shape (SS) cues, which arise due to the direction-dependent filtering characteristics of the head and pinnae [16]. Although SS cues are generally considered less essential for horizontal sound localization than ITD and ILD cues [17], identical or nearly identical ITD and ILD cues occur at forward and mirror-opposite rearward azimuths. Disambiguation of forward from rearward (“front” from “back”) source azimuths therefore *requires* the combination of ITD/ILD and SS information [16,18]. SS cues are also important for localization of sources in elevation, as variations across ITD and ILD in elevation (at a fixed azimuth) are negligible [19]. However, HPDs partially or fully occlude the external ear canal and/or concha, and can thus significantly distort SS cues [20,21]. Consequently, subjects tested in auditory spatial tasks during HPD use exhibit high rates of “front-back” and “up-down” errors [8]. Reduced ability to localize sound sources is detrimental to situational awareness, and may threaten survivability in high-risk situations [22]. In combination, reduced situational awareness and audibility, and physical discomfort can discourage at-risk personnel from HPD use [10,11]. The development of comfortable HPDs that protect hearing without compromising auditory perceptual abilities could thus dramatically improve safety and reduce the prevalence of hearing loss in military and industrial settings. Devices with smaller in-ear profiles that result in less occlusion of the concha while still providing effective attenuation, for example, might cause less distortion of critical SS cues and result in fewer front-back confusions.

The experiments described in this report were designed to measure the effect of four different HPDs (two active, two passive) on (1) sound source localization, (2) speech recognition, (3) and tone detection in a group of young, normal-hearing subjects. Psychophysical measurements were compared to measurements of head-related transfer functions made using a binaural test manikin with and without tested HPDs in place [8]. Collectively, data demonstrated minor decrements in tone detection and speech recognition ability during HPD use, but marked increases in sound localization errors that were strongly predicted by the degree of SS cue distortion produced by each of the four devices.

## Methods and Materials

### Ethics Statement

All testing complied with ethical guidelines set forth by the National Institutes of Health of the United States of America. Written informed consent was obtained from all subjects prior to the commencement of testing. All consent and testing procedures were specifically approved by the Colorado Multiple Institutional Review Board under protocol # 13–3043.

### Subjects

Subjects were young adult males (mean 29.5 yrs ± 4.8 sd, *n =* 10) with normal hearing defined by audiometric thresholds ≤25 dB HL bilaterally at octave frequencies over the range 250–8000 Hz. Prior to testing, subjects’ ear canals were inspected via otoscope for cerumen buildup or other contraindications to HPD insertion.

### Hearing protection devices

Three HPDs were used in all tests. A fourth was used in sound localization testing only. Devices were:

1. The 3M Combat Arms (3M, St. Paul, MN). This device is a passive HPD with two listening modes. The “open” mode is designed to suppress high amplitude impulsive events by utilizing a nonlinear passive acoustic filter element [12]. The “closed” mode is designed to provide maximum protection against continuous noise. In this study, the device was tested in “open” mode only.
2. The Etymotic EB15 (Etymotic, Elk Grove Village, IL). This device is an active, battery-powered device with a microphone and speaker. The microphone is located on the lateral side of the device near the forward edge. The device has two listening modes. The “high” mode provides 15 dB of gain above natural hearing below 60 dB SPL source level, decreasing ~linearly to 0 dB of gain above natural hearing above 90 dB SPL source level. The “low” mode provides 0 dB of gain below 60 dB SPL source level decreasing ~linearly to 15 dB of attenuation below natural hearing above 80 dB SPL source level. In this study, this device was tested in “high” mode only.
3. A prototype active device similar in concept to the EB15, hereafter referred to as the “Hybrid” device. The Hybrid is slightly larger in profile (extending further laterally and vertically in the concha) than the EB15. Like the EB15, this device is an active, battery-powered device with a microphone and speaker. The microphone is located at the end of a narrow-diameter channel on the forward-facing side of the device. This device also has “high” and “low” listening modes. The “high” mode is designed to provide 0 dB of attenuation (i.e., no attenuation) up to a 110 dB SPL source level. The “low” mode is designed to provide 20 dB of attenuation relative to normal hearing up to a 110 dB SPL source level. In this study, the device was tested in “high” mode only.
4. A prototype passive device similar in concept to the Combat Arms device, hereafter referred to as the “ShotShields” device. This device is considerably smaller in profile (extending only a few millimeters into the concha from the ear canal) than the Combat Arms device. Like the Combat Arms device, the ShotShields has two listening modes. The “open” mode is designed to suppress high amplitude impulsive events by utilizing a nonlinear passive acoustic filter element (a single pinhole with a diaphragm element, rather than the double pinhole in the Combat Arms device). The “closed” mode is designed to provide maximum protection against continuous noise. In this study, this device was tested in “open” mode only.

**Fig 1:**
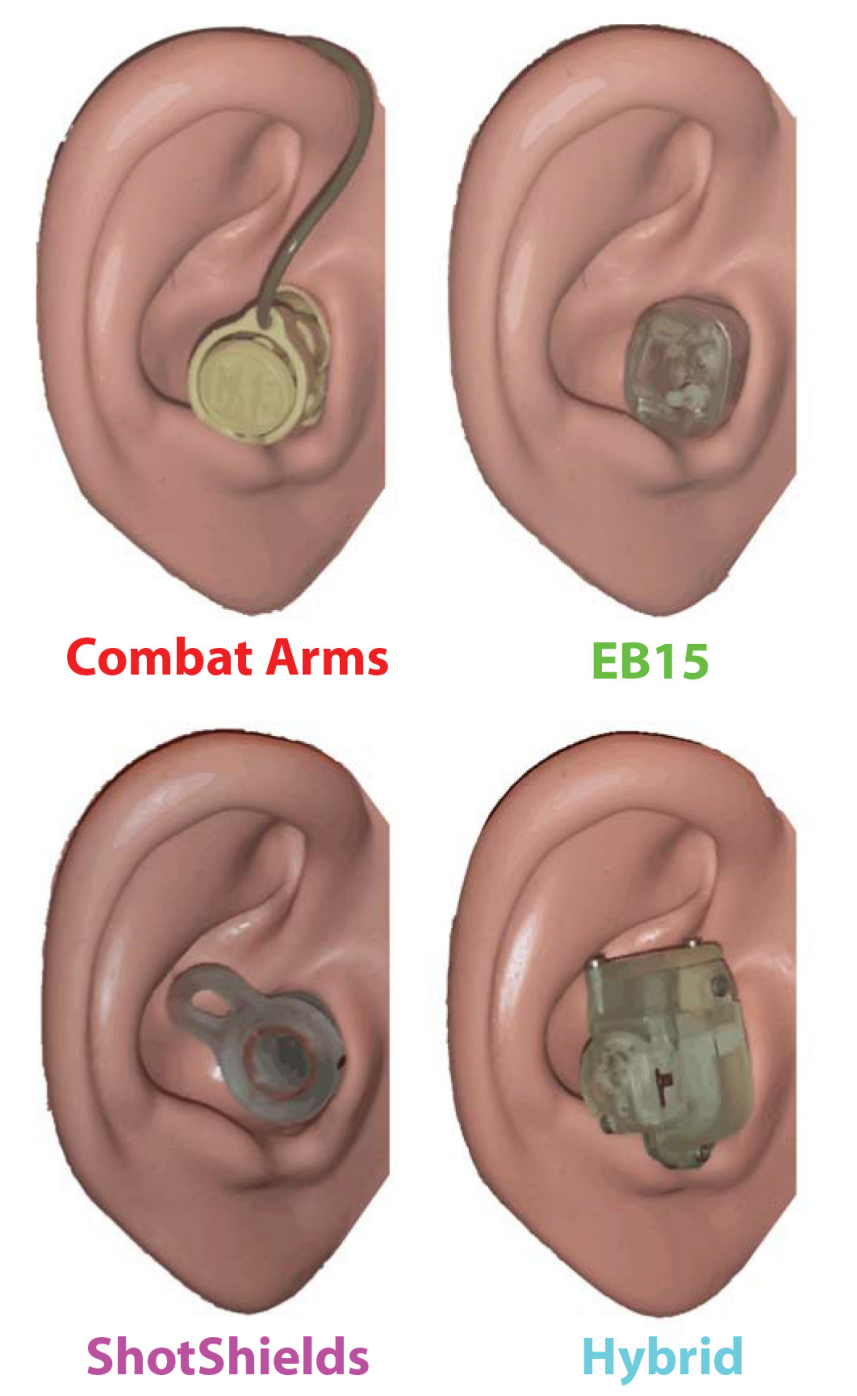
Hearing protection devices (HPDs) tested in the current study. Four HPDs are illustrated as worn inside the right ear of a test manikin (G.R.A.S. 45CB Acoustic Test Fixture).

The Combat Arms device, which has silicon insert tips, was cleaned with alcohol prep pads between testing sessions. The other devices had removable foam tips, with a set of tips allocated to each subject for repeated use across sessions (“medium” non-custom tips fit all subjects comfortably and were used across experiments). Batteries (type 312) in active devices were replaced at regular weekly intervals. All four devices are illustrated in Fig 1, placed inside the right pinna of an ANSI standard test manikin (45CB Acoustic Test Fixture, G.R.A.S. Sound & Vibration A/S, Holte, Denmark).

### Experimental procedures

#### Common procedures

**Fig 2:**
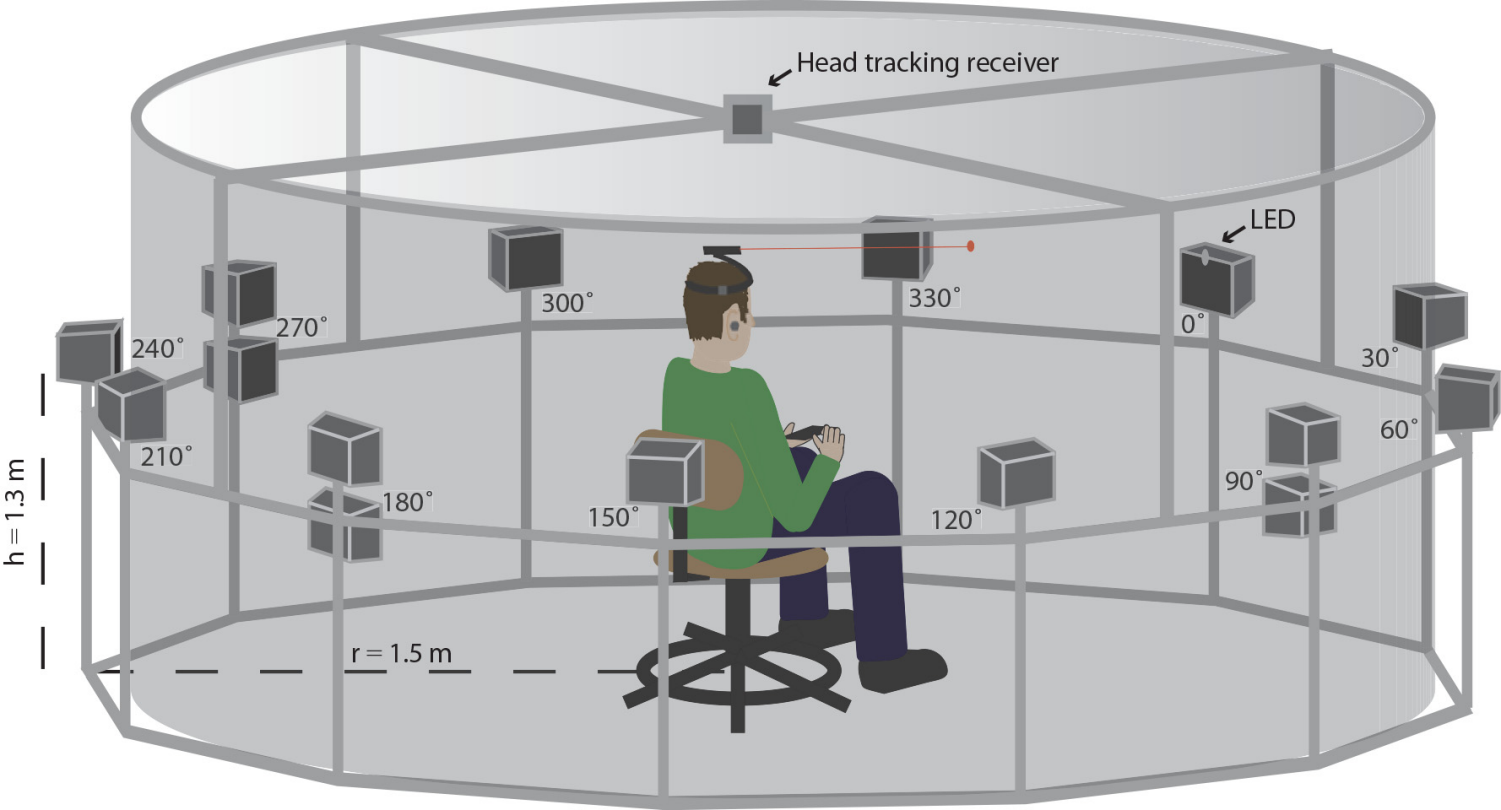
Testing apparatus used in all three psychophysical tasks. Subjects were seated in a circular array of loudspeakers and fitted with a headband supporting (1) an electromagnetic position sensor and (2) a laser pointer. Speakers were obscured by an acoustically transparent curtain, and all testing was completed in total darkness. An LED demarcated the nominal 0° loudspeaker, which served as the starting position on each trial in the localization task, was the target loudspeaker in tone detection and speech-in-noise tasks. Head position was monitored continuously via the overhead position-tracking receiver. See text for additional details.

All testing was completed in structurally isolated 5 m × 5.5 m × 3 m room (broadband RT_60_ ≈ 0.5 s). The testing apparatus, located at the center of the room, consisted of 12 loudspeakers (Pyle PDWP5BK, Brooklyn, NY) mounted ~1.3 m from the floor in a circular array (radius ~1.5 m) at 30° intervals (12 speakers total). The speakers and mounting posts were obscured from view by an acoustically transparent curtain. Testing was completed in total darkness to further limit the use of visual cues. A PVC frame was affixed to the speaker array to support an electromagnetic position-tracking receiver (Polhemus Fastrak, Colchester, VT) at its center. A height-adjustable stool, on which the subject sat, was located directly below the position tracker. During all testing, subjects sat on the stool and wore an adjustable plastic headband with a laser pointer mounted along its midline and a position-tracking sensor (Polhemus Fastrak) mounted at its side. The height of the stool was adjusted as needed to bring the speakers to ear level; head position was calibrated for each subject at the beginning of each testing session, or any other time the headband was adjusted, by using the “boresight” feature of the Polhemus Fastrak system. Specifically, subjects were instructed to align the laser pointer projection with an LED affixed to the nominal 0° loudspeaker, adjust the headband as needed, and press a handheld button when the laser pointer and LED were aligned, both at the center of neutral gaze. In all tasks, testing began only when the subject was facing forward, ±5° in azimuth and elevation. The testing apparatus is illustrated in Fig 2. All stimuli were synthesized at 44.1 kHz using a personal computer soundcard (RealTek, Taiwan), amplified (Crown XTi 4000, Elkhart, IN), and relayed to the target speaker via a multiplexer controlled with a digital I/O board (National Instruments USB-6501, Austin, TX). Stimulus design and task varied across experiments, as detailed in the following sections. Stimulus presentation and data acquisition were controlled with custom software developed in MATLAB (MathWorks, Natick, MA).

#### Sound source localization paradigm

Stimuli were recordings of a cocking AK-47 (a loud “click-click”; a military-relevant and easily-localizable broadband signal used in previous HPD localization studies, with usable ITD, ILD and SS cues, approximately 1 s in total duration) presented at an average level of 70 dB SPL. The stimulus level was varied randomly over a ±5 dB range from trial-to-trial to minimize the use of overall level cues in sound location judgments, e.g., due to small variations in subject head position toward or away from target speakers over the course of testing. The chosen range of levels was sufficient to be plainly audible by all subjects with all HPDs, while avoiding any peak-limiting HPD features. Additionally, broadband masking noise was produced by a function generator (Agilent 33220A, Santa Clara, CA) and presented continuously at 55 dB SPL from three loudspeakers (Pyle PDWP5BK, Brooklyn, NY) mounted just below the target speakers at +90°, -90° and 180° azimuth.

Sound localization accuracy was tested in a single-interval orienting paradigm [23,6]. On each test trial, a target stimulus was presented from a randomly selected loudspeaker. The subject was instructed to turn his head to point the laser projection at the perceived sound source location. The response azimuth, recorded by the position tracking system when the subject pressed a handheld button (with ~±0.1° of error), was computed relative to the measured forward position to account for any variations in headband placement across sessions or subjects. If subjects did not return to the forward position within 2 seconds after the conclusion of a trial, the LED at 0° was illuminated until the subject returned to the forward position.

Testing was completed in sets of 120 trials (10 trials per each of 12 loudspeakers). After an initial unoccluded training period generally lasting <2 h (subjects completed on average ~3 training runs in order to familiarize themselves with the apparatus and paradigm), subjects were tested in an unoccluded (Control) condition, and in the 4 HPD conditions. Each subject completed three sets of trials for each condition, i.e., 30 trials for each speaker within each condition. The order of device testing was varied across subjects using circular permutation to reduce order effects at the group level, except that all subjects completed testing with the ShotSheild device at the end of the study, as this prototype device became available for study after testing with the other 3 HPDs had already commenced.

#### Speech-in-noise paradigm

Subjects’ ability to understand speech in the presence of interfering talkers was tested using a modified version of the widely used and clinically validated QuickSIN test [24]. QuickSIN stimuli consist of pre-recorded lists of 6 short sentences, spoken by a female, each containing 5 “target” words (e.g. “It is a band of steel three inches wide” or “Crouch before you jump or miss the mark”) presented in a multi-talker background. The test is intended to simulate the everyday challenge of understanding a talker in a crowded social setting. Following each sentence, the subject is instructed to repeat as many words as possible back to the experimenter. The test becomes progressively more difficult with each sentence, because the signal-to-noise ratio is decreased in 5 dB steps from +25 dB for the first sentence to 0 dB for the last sentence. Performance is scored for each list according to the number of target words (out of 30) correctly identified. Modifications for use of the QuickSIN in the current project were as follows: (1) the target sentence was presented at a level of approximately 65 dB SPL from the forward (0°) speaker, while the multi-talker background noise was presented from 3 loudspeakers located at ±90° and 180° (in clinical settings, stimuli are typically presented through a single loudspeaker directly in front of the listener, or over headphones with the target and interferers presented separately to each ear), (2) all signal-to-noise ratios were decreased by 3 dB, such that the first sentence of each list was +22 dB signal-to-noise and the last was -3 dB (rather than +25 to 0). Both manipulations were designed to make the task more difficult. Subjects completed 3 lists for each of 4 conditions (Control, and all HPDs except the ShotShields), thus including all 12 “two-channel” recordings in the QuickSIN battery), with the same lists assigned to each device across subjects, but the order of device testing again varied across subjects. The final score for each subject per condition was taken as the average score (out of 30) across the 3 tested lists. (Note: in clinical testing, scores are normalized to give “signal-to-noise loss,” a measure of impairment; this step is unnecessary for our cross-HPD comparisons).

#### Tone detection paradigm

Stimuli were pure tones of octave frequencies from the range 250–8000 Hz (6 different frequencies). All tone detection stimuli were presented from the forward (0° azimuth) loudspeaker in the array illustrated in Fig 1. Head position was monitored continuously to ascertain that the subject maintained a forward orientation (±5°) over the duration of each session. Presentation level, the stimulus parameter of primary interest, was varied adaptively from trial-to-trial using a two-alternative forced-choice procedure, as follows:

Each trial consisted of two consecutive presentation intervals, each signaled by a 500-ms duration light burst from the LED affixed to the forward loudspeaker. Immediately following *one* of these light bursts (determined randomly), a 500-ms duration tone burst was presented from the forward (0°) loudspeaker. The two intervals were separated by a 1-s pause (during which neither light nor sound was presented). The subject’s task was simply to indicate whether the tone had been presented during the first or second interval (or to guess if uncertain) by pressing a glowing “1” or “2” button on a handheld keypad.

Each testing session began with presentation of a 40 dB SPL tone (easily detectable in all conditions for all subjects). Tone level was then varied according to the subject’s response on the preceding trial using a 3-down, 1-up rule: tone level was lowered by 8 dB following 3 correct responses (a downward reversal), or increased by 8 dB following 1 incorrect response (an upward reversal). After the fourth overall reversal, the step size was decreased from 8 dB to 1.5 dB. The session was terminated after the 8^th^ overall reversal, and the tone detection threshold was taken as the average level at the final 4 reversals to estimate the stimulus level giving 79.4% correct discrimination [25] for each frequency-HPD combination. Two thresholds were measured in two separate sessions for each frequency-HPD combination. As for localization and speech-in-noise tasks, the order in which HPDs were tested was varied across subjects via circular permutation. Within-subjects, the order of tested frequencies was randomized for both the first and second runs. Subjects completed runs of training at 250, 1000 and 8000 Hz (1 run each frequency) without HPDs prior to beginning the experiment to familiarize themselves with the task, and were given regular breaks during all subsequent testing sessions.

#### Device comfort ratings

Finally, at the conclusion of testing, subjects were asked to rate the comfort of the 3 HPDs included in all tests (Combat Arms, EB15, Hybrid) by completing a brief questionnaire. Each questionnaire (one per device) consisted of 3 questions: (1) “How comfortable was this device for physical fit?” (2) “How comfortable was this device for listening comfort?” and (3) “Considering everything, how much did this device change your enjoyment of listening?” Subjects responded to each question using a Likert scale (1 = Very Uncomfortable, to 5 = Very Comfortable), giving a worst possible device comfort score of 3 and best possible score of 15.

### Statistical methods

Quantification of performance for statistical analyses was straightforward for the tone detection, speech-in-noise, and comfort rating tasks. Quantification of localization accuracy for statistical analysis required the derivation of useful accuracy measures. Particularly in localization tasks that produce a large number of front-back errors (e.g., localization with HPDs), it is not sensible to quantify accuracy in terms of “mean response” (e.g., if half of a subject’s responses were toward 0° and the other half toward 180°, the mean would be 90°, even though the subject never once responded near 90°). Thus, it is more useful to quantify localization error directly. We employed two such measures that treated performance differently.

First, we calculated root-mean-square (RMS) error, which quantified the mean absolute error in degrees across trials for each speaker location (i.e., the mean angular distance of responses from the target angle). Higher RMS error for a given target location indicates poorer accuracy. RMS error is useful for measuring relatively small changes in accuracy that may result, for example, from slight distortions of ITD or ILD cues. However, the difference between an RMS error of 15° and 10° in single-interval localization task, where all responses cluster around the target location, may not be of especially high ecological significance; in either case, the observer would have turned his head roughly toward the source.

Thus, second, we calculated the incidence of Very Large Errors (VLEs)–errors greater than 45°. VLEs lead the subject to turn his head in the wrong direction (e.g., toward 120° for a target at 60°, toward 150° for a target at 30°, or toward 180° for a target at 0°). Such errors are generally “front-back” confusions, arising due to distortions of SS cues, but could also arise due to severe ITD or ILD distortions leading to gross mislocalization (e.g., a response of 80° for a target at 30°). Such errors are of high ecological significance because they also misdirect the visual system. VLEs were calculated for each speaker location by summing the total number of errors larger than 45° occurring for that location, again for each device by subject combination. Both VLEs and RMS were averaged across subjects and are plotted in addition to single subject data. “Grand mean” RMS and VLE measures were also computed, according to the mean RMS error or VLE total across all source angles.

Statistical differences within all tasks were assessed across conditions using conventional null-hypothesis testing with a criterion alpha level (i.e., maximum *p*-value for significance) of 0.05, implemented in either MATLAB or SPSS (v17.0, SPSS, Inc.). Tests included repeated-measures (i.e., within-subjects) ANOVA and paired *t*-tests. The sometimes-large number of paired comparisons (up to 10) should be noted when evaluating reported *t*-test *p*-values in terms of statistical significance; family-wise Bonferroni correction (in this instance, simply 0.05 divided by the number of paired comparisons in the set) gives a conservative endpoint. Nonetheless, exact *p*-values are reported throughout the text (unless below 0.001, in which case <0.001 is indicated).

## Results

### Sound source localization

**Fig 3:**
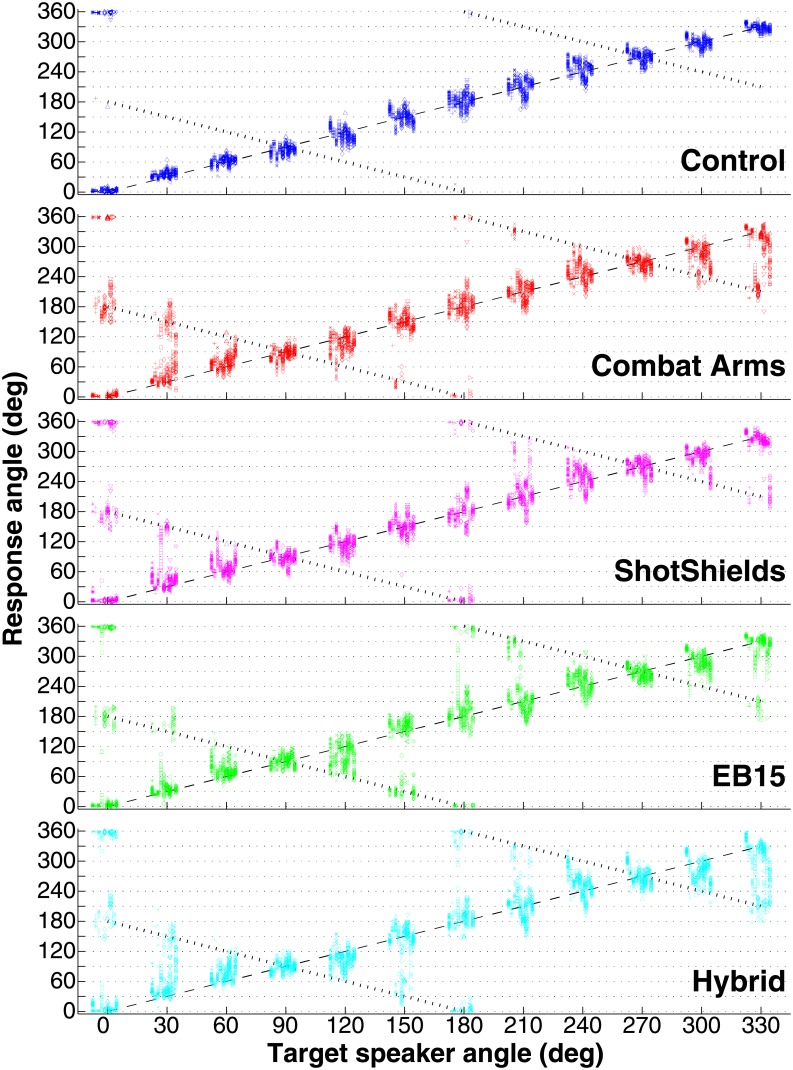
Localization responses across 5 conditions (Control and 4 HPDs). Symbols are shifted along the abscissa around each target angle for clarity. The dashed black line (unity) indicates perfect performance. Dotted black lines with negative slopes indicate responses expected for trials on which subjects experienced front-back confusions (see text).

Fig 3 plots raw localization data (response angle as a function of target speaker) for all 10 subjects across the five test conditions (Control, 4 HPDs). Individual subject responses are offset slightly along the abscissa so that cross-subject variability is easier to visualize. Perfect performance is indicated by the dashed black line (unity). Subjects were generally very accurate in the Control condition, with almost all responses clustered near unity (responses just below 360° for a target at 0° are near unity in a circular sense and yield RMS values near 0°). Concordant with previous work [23], accuracy was generally somewhat higher for forward than for lateral or rearward sources. Importantly, mean accuracy in the baseline condition was also comparable overall to that reported in previous studies wherein testing was conducted inside anechoic rooms [26], suggesting negligible room effects in the present study (i.e., no evidence for performance degradations due to echoes and reverberation).

**Fig 4:**
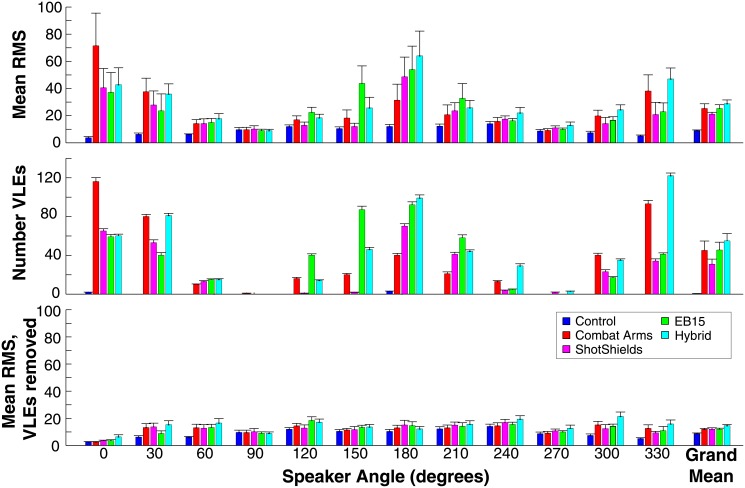
Mean localization errors across subjects. Mean root-mean-square errors (RMS; upper panel, lower panel) or very large errors (VLEs; middle panel) are given for each response angle across conditions. The lower panel gives RMS values after removal of trials on which VLEs occurred (see text). A grand mean is also given across devices for each measure, based on each subject’s mean error across target speaker angles. Error bars indicate ± 1 standard error across subjects.

HPD use gave rise to markedly increased response variability in most subjects, including the emergence of multiple clusters of responses for single source locations (e.g., responses clustered both near 0°/360° and 180° for targets at 180° or 0°). Worst accuracy generally occurred for forward and backward locations, apparently the result of front-back confusions (dotted lines), while best accuracy occurred for lateral locations (90° and 270°). Fig 4 plots mean total RMS error (upper panel), VLE incidence (middle panel), and RMS error after removal of VLE trials (lower panel) across target angles and conditions and overall (far right of each panel; see legend). To assess statistical differences across conditions, both RMS and VLE measures were submitted to 5x12 repeated-measures (within-subjects) ANOVAs with factors of device condition and target angle. The results of these analyses and follow-up paired tests are reported in the following sections.

#### Analysis of RMS error

The ANOVA for RMS error indicated a significant main effects of device condition (*F*_4,36_ = 13.64, *p*<0.001) and target angle (*F*_11,99_ = 5.67, *p*<0.001), and a significant interaction between device and target angle (*F*_44,396_ = 1.87, *p* = 0.001), which appeared to be carried by the clear divergence of Control and HPD conditions, most especially at forward and rearward target angles. To assess differences across HPDs in more detail, a series of post-hoc paired comparisons was conducted. Comparisons were blocked across target angles. Only forward (330°, 0° and 30°) and rearward (210°, 180° and 150°) targets were included in the analysis. Results, starting at 0° and moving clockwise were as follows:

For a **0**° **target**, the Combat Arms (*t*_9_ = 2.78, *p* = 0.021), ShotShields (*t*_9_ = 2.69, *p* = 0.25), EB15 (*t*_9_ = 2.28, *p* = 0.049) and Hybrid (*t*_9_ = 3.07, *p* = 0.013) devices were all worse than Control, but not significantly different from each other (lowest *p* = 0.179). For a **30° target**, the Combat Arms (*t*_9_ = 3.33, *p* = 0.009) and Hybrid (*t*_9_ = 4.04, *p* = 0.003) devices were significantly worse than Control, while the ShotShields (*t*_9_ = 2.18, *p* = 0.057) and EB15 (*t*_9_ = 1.41, *p* = 0.192) were not. None of the devices were significantly different from each other (lowest *p* = 0.234). For a **150° target**, the EB15 (*t*_9_ = 2.74, *p* = 0.023) was worse than Control, and also worse than the ShotShields (*t*_9_ = 2.49, *p* = 0.035) and Combat Arms (*t*_9_ = 2.32, *p* = 0.045). No other devices were significantly different from each other or from Control (lowest *p* = 0.089). For a **180° target**, the ShotShields (*t*_9_ = 2.60, *p* = 0.029), EB15 (*t*_9_ = 2.37, *p* = 0.042) and Hybrid (*t*_9_ = 2.86, *p* = 0.019) were worse than Control. No other devices were significantly different from each other or from Control (lowest *p* = 0.091). For a **210° target**, the Hybrid (*t*_9_ = 2.65, *p* = 0.027) was worse than Control. No other devices were significantly different from each other or from Control (lowest *p* = 0.074). For a **330**° **target**, the Combat Arms (*t*_9_ = 2.70, *p* = 0.025), EB15 (*t*_9_ = 2.65, *p* = 0.026) and Hybrid (*t*_9_ = 4.84, *p* = 0.001) were all worse than Control. The Hybrid was also worse than the EB15 (*t*_9_ = 2.76, *p* = 0.022), and marginally worse than the ShotShields (*t*_9_ = 2.24, *p* = 0.052). No other devices were significantly different from each other (lowest *p* = 0.111).

To assess pair-wise differences in overall RMS error, RMS error was averaged across target angles within subjects to create a new measure, “grand mean” RMS error. Paired-tests were then conducted on these values (given at the far right of Fig 4, upper panel). In this analysis, the Combat Arms (*t*_9_ = 4.44, *p* = 0.002), ShotShields (*t*_9_ = 8.63, *p*<0.001), EB15 (*t*_9_ = 5.40, *p*<0.001) and Hybrid (*t*_9_ = 6.47, *p*<0.001) were clearly all worse than Control. Among HPDs, the ShotShields had the lowest grand RMS, though the difference approached significance only for the comparison against the Hybrid device (*t*_9_ = 2.42, *p* = 0.039).

#### Analysis of VLE incidence

The ANOVA for VLE incidence, like that for RMS error, indicated significant main effects of device condition (*F*_4,36_ = 14.81, *p*<0.001) and target angle (*F*_11,99_ = 5.08, *p*<0.001), and a significant interaction between device and target angle (*F*_44,396_ = 1.87, *p*<0.001), again carried by the clear divergence of Control and HPD conditions, most especially at forward and rearward target angles. In fact, VLEs almost never occurred in the Control condition, with 5 over the course of the entire experiment (out of 3600 Control trials; a rate of ~0.1%). To gain more detailed information on VLE increases caused by HPDs, pair-wise tests were again conducted in a block-wise fashion for forward and rearward target directions. Results did not differ materially from those reported for RMS error. Notably, “grand mean” VLE (far right of middle panel in Fig 4) also demonstrated that the Combat Arms (*t*_9_ = 4.53, *p* = 0.001), ShotShields (*t*_9_ = 5.69, *p*<0.001), EB15 (*t*_9_ = 5.52, *p*<0.001) and Hybrid (*t*_9_ = 7.17, *p*<0.001) were all worse than Control, while the ShotShields produced the fewest VLEs and significantly fewer than the Hybrid device (*t*_9_ = 2.95, *p* = 0.016).

#### Analysis of RMS error excluding VLE trials

Despite their different derivations and theoretical distinction, the close parallel of RMS and VLE measures in the present dataset is not surprising. Although RMS can be elevated without producing any VLEs (e.g., 0° vs. 90° in the Control condition), the majority of HPD-related RMS elevation in the present dataset appears to be attributable to VLEs due to front-back confusions [6]. To analyze RMS error *apart* from VLEs, we recalculated RMS after explicitly excluding trials on which errors were larger than 45°. These values are plotted, averaged across subjects, in the lower panel of Fig 4. RMS errors were generally of a much-reduced magnitude after VLE removal in HPD conditions, but were still somewhat elevated relative to control. To assess these differences statistically, grand mean RMS values (lower panel of Fig 4, far right) were submitted to pair-wise testing. The Combat Arms (*t*_9_ = 3.43, *p* = 0.008), ShotShields (*t*_9_ = 3.17, *p* = 0.11), EB15 (*t*_9_ = 2.82, *p* = 0.020), and Hybrid (*t*_9_ = 5.75, *p*<0.001) were all worse than Control. The Hybrid was also worse than the EB15 (*t*_9_ = 3.10, *p* = 0.014), and Combat Arms (*t*_9_ = 2.74, *p* = 0.023).

Data thus suggest that HPDs cause both large errors (VLEs, i.e., front-back confusions), detected in a majority of HPD localization studies, as well as smaller errors (i.e., increased “localization blur”), perhaps due to distortions of ITD or ILD cues, or reduced interaural correlation [16]. Nonetheless, as VLEs are clearly the dominant source of localization error during HPD use, the remainder of our analysis focuses on RMS error including VLEs, or on VLEs exclusively.

#### Localization results summary

In sum, all tested HPDs significantly degraded localization performance relative to Control. Though some differences between HPDs emerged at particular source angles, no mean (cross-azimuth) differences were evident. In absolute terms, the ShotShields device produced the lowest mean RMS error and lowest mean incidence of VLEs, followed (in ascending/worsening order) by the Combat Arms, EB15, and Hybrid. It is possible that practice effects contributed to lower mean errors for the ShotShield, as this device was not integrated into the counter-balanced experimental design, and was tested last in all cases [9]. However, post-hoc analyses of HPD-related distortions of SS localization cues demonstrated that the ShotShields produced both the best behavioral performance and the greatest preservation of SS cues (see Discussion).

**Fig 5:**
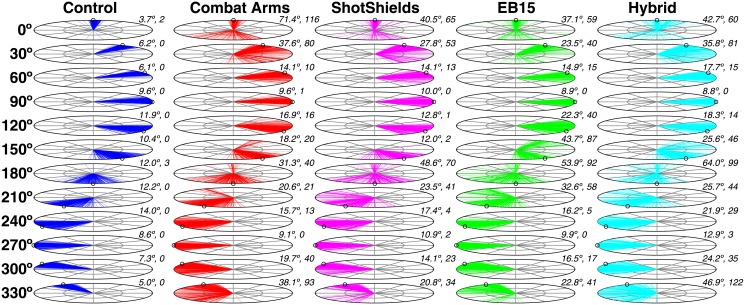
Localization data across all source angles and device conditions, plotted in polar coordinates. Responses from all subjects are combined on the same axes. Each radius (colors) gives the head turn response angle for a single trial. Open circles around the perimeter of each plot indicate the target azimuth. The mean response error across subjects (RMS) and number of VLEs (out of 300 possible), respectively, are shown to the upper right of each plot.

Fig 5 plots data from all conditions in polar coordinates so that the data may be visualized more easily with respect to the testing array. Each radius gives a single head turn response, with all trials for all tested subjects combined on the same target angle × condition axes (on each plot, the target location is indicated by an open black circle on the perimeter). The numbers to the upper right of each plot give the mean (cross-subject) RMS and total number of VLEs (out of 300 possible, with 300 trials per angle per HPD condition across subjects).

### Speech-in-noise recognition

**Fig 6:**
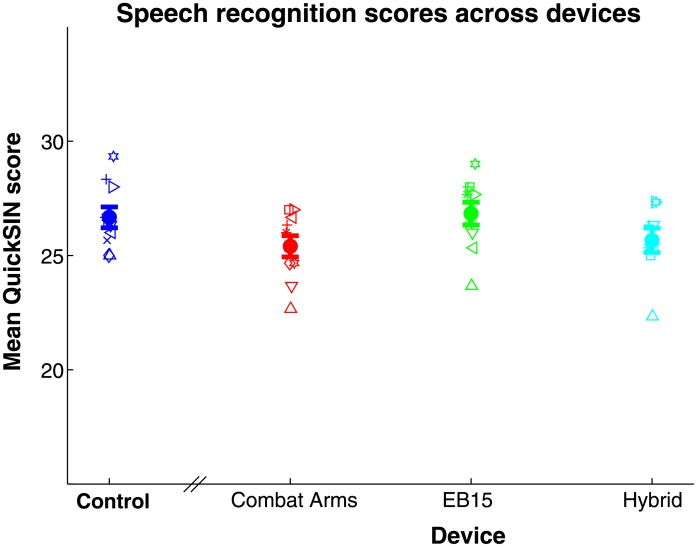
Mean QuickSIN score across device conditions. Higher numbers indicate better performance. Symbols give data for individual subjects; bold points give the mean across 10 subjects ±1 standard error.

Fig 6 plots mean QuickSIN scores (representing the average number of correctly identified target words out of 30, across 3 sentence lists for each condition) for all subjects (symbols) and cross-subject means (bold filled circles, ±1 standard error). To assess differences in speech recognition across HPD conditions, scores were submitted to a one-way (device condition, 4 levels) repeated-measures ANOVA. The main effect of HPD condition was significant (*F*_3,27_ = 5.88, *p* = 0.003), accounting for 40% of the variance in scores across conditions (*partial η* ^*2*^ = 0.40). Follow-up pair-wise tests revealed that performance was significantly worse than Control for the Combat Arms (*t*_9_ = 3.00, *p* = 0.015) and Hybrid (*t*_9_ = 3.53, *p* = 0.006), while performance for the EB15 was very similar to Control performance (*p* = 0.595), and better than performance with the Combat Arms (*t*_9_ = 2.41, *p* = 0.039) or Hybrid (*t*_9_ = 3.17, *p* = 0.011). Thus, the EB15 provided the best speech recognition among the 3 HPDs included in the speech-in-noise task (perhaps related to better audibility at high frequencies; see below), although mean scores for all conditions were above 25, better than 80% correct.

### Tone detection

**Fig 7:**
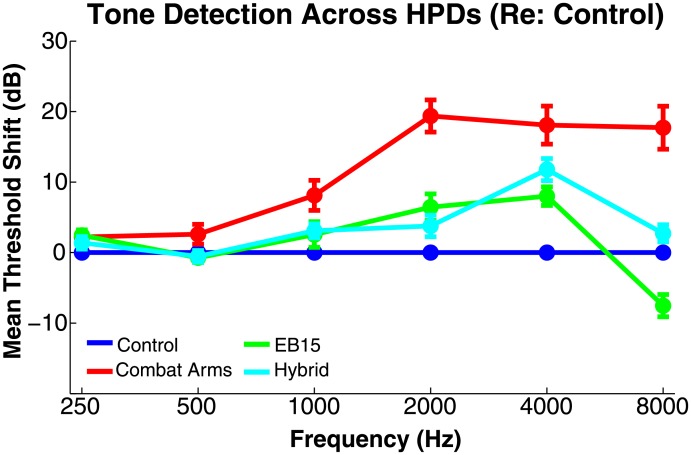
Tone detection thresholds across frequency (abscissa) and HPDs (parameter) relative to Control. Lower numbers indicate better performance. Bold points give the mean across 10 subjects ±1 standard error.

Tone detection thresholds in the unoccluded (Control) condition generally followed the cross-frequency trajectory expected on the basis of previous reports [27]. As we were concerned with HPD-related *changes* in thresholds, Fig 7 plots tone detection thresholds obtained with HPDs in place normalized to thresholds obtained in the Control condition (i.e., Control thresholds were subtracted from HPD thresholds on a per-subject basis). Thresholds are plotted as means ±1 standard error; individual subject data followed the trends evident in the mean data. The most notable elevation of thresholds (decrement in sensitivity) occurred for the Combat Arms device. The EB15 and Hybrid also deviated from Control performance at several frequencies, with the EB15 mean threshold falling *below* the Control threshold at 8 kHz.

Threshold differences were assessed statistically using raw (dB SPL) threshold values via a 4x6 (device condition × frequency) repeated-measures ANOVA. The main effects of device (*F*_3,27_ = 33.87, *p*<0.001) and frequency (*F*_5,45_ = 137.60, *p*<0.001) were both significant, as was the device × frequency interaction (*F*_15,135_ = 212.26, *p*<0.001). To assess differences across devices in more detail, threshold values were submitted to pair-wise testing, conducted in a block-wise fashion at the 4 frequencies (1000–8000 Hz) where inter-HPD differences appeared largest. Differences across HPDs and between HPDs and Control were generally significant where points in Fig 7 do not overlap (e.g., at 4000 Hz, all HPDs were significantly worse than Control, and significantly different from each other, with the EB15 performing best, followed by the Hybrid and the Combat Arms. Notably, at 8000 Hz, the EB15 was *better* than Control (*t*_9_ = 4.77, *p* = 0.001), the Combat Arms (*t*_9_ = 12.46, *p*<0.001), and the Hybrid (*t*_9_ = 16.25, *p*<0.001). In sum, data demonstrate larger reductions in tone detection ability for the passive Combat Arms device than for the two active devices, but significant reductions (particularly at mid-frequencies) for all three HPDs included in the tone detection task.

### Device comfort ratings

**Fig 8:**
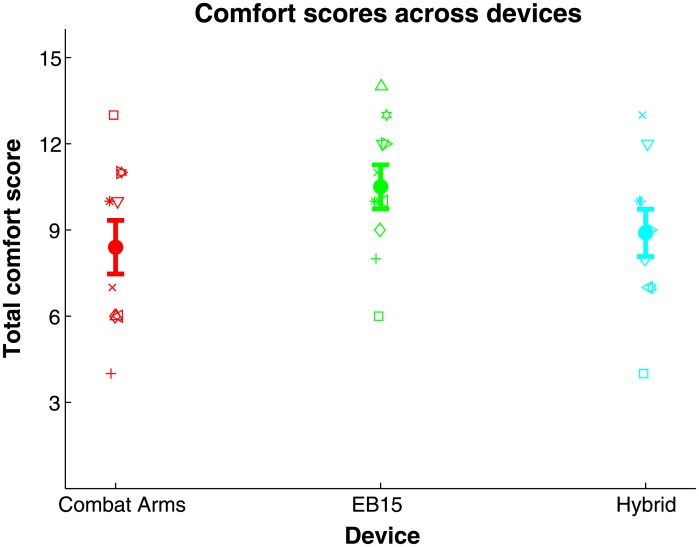
Comfort ratings across the 3 devices tested used in all tests (Combat Arms, EB15, Hybrid). Bold points give the mean across 10 subjects ±1 std. error.

Finally, Fig 8 plots device comfort rating scores (see Methods) across the three HPDs used in all three perceptual tests (symbols), and the cross-subject mean for each (bold circles, ±1 standard error). The cross-subject mean was worst for the Combat Arms device, followed by the Hybrid, then the EB15. Inter-subject variability was high, with no consistent cross-device differences across subjects.

## Interim Discussion

Three different auditory perceptual tests were administered to a group of young normal-hearing males to assess the effects of HPD use on (1) sound source localization accuracy, (2) speech recognition, and (3) tone detection. Additionally, a questionnaire was administered to assess the physical and listening comfort of the tested HPDs. The major novelty of the present study was its assessment of performance with new and previously untested HPDs. The study was also unique in its measurement of multiple auditory perceptual capacities across the same HPDs in the same group of subjects [7], and in its precise measurement of localization accuracy *per se* using an ecologically valid response paradigm [6]. Concordant with previous reports, HPD use significantly degraded sound localization [5,6,8] and signal detection [7,12]. Speech recognition was also somewhat degraded, although one HPD (the EB15) did not significantly change performance. No significant differences in subjective comfort were reported across HPDs, although some subjects had clear preferences for one HPD over the others. Anecdotally, several subjects described the Combat Arms device as “itchy” inside the ear canal. Several subjects also expressed frustration at feedback (“whistling”) produced by the Hybrid device during insertion.

The most notable negative perceptual effect of HPD use was the marked increase in large localization errors (VLEs), attributable to confusions of front and back locations (readily visualized in Fig 5). While we found no significant differences in RMS values or VLE rates across HPDs (all were significantly worse than unoccluded performance), the ShotShields—a previously untested passive device—produced the lowest mean RMS error and the lowest mean number of VLEs. The Hybrid—the previously untested active device—gave the worst overall performance, while the Combat Arms and EB15 devices produced an intermediate number of errors. Toward a deeper understanding of the incidence of front-back confusions with the tested HPDs, we completed acoustic measurements using a test manikin fitted with the tested HPDs and attempted to relate patterns in acoustic distortions caused by HPDs to patterns in psychophysical localization data [8].

## Comparison of Acoustic Distortions and Localization Errors

### Measurement of head-related transfer functions

#### Experimental setup

Head-related transfer functions (HRTFs) characterize the effect of the head, torso, and external ears on impinging sound [17]. In the context of sound localization, SS cues (spectral shape; see Introduction) are simply the prominent features of the HRTF that provide information about sound source location. While SS cues are generally considered most important for localization in elevation, which cannot be achieved using interaural (ITD and ILD) cues alone, SS cues are also useful for resolving forward from rearward locations [19]. Therefore, in order to characterize the effect of the HPDs we tested on SS cues, we measured HRTFs for all tested HPDs using an acoustic test manikin designed specifically for the testing of HPDs (G.R.A.S. 45CB Acoustic Test Fixture, see ANSI S12.42).

Stimulus generation and data acquisition procedures were similar to those described in previous publications from our laboratory [28]. Briefly, all experiments were performed in a ~3 × 3 × 3 m double-walled sound-attenuating chamber (IAC, Bronx, NY) lined on all 6 inner surfaces with acoustic foam. Stimuli were presented from 25 identical loudspeakers (Morel MDT-20) attached to a custom-built semicircular boom with radius of 1 m. The 25 loudspeakers were spaced along the boom at 7.5° intervals, from +90° (left) to -90° (right). The interaural axis of the test manikin was aligned with the axis of rotation of the boom using mounted laser pointers. Recordings were made from a total of 775 different locations, covering azimuth (+90° to -90°) and elevation (-45° in the frontal hemisphere to +180°, exactly behind the manikin). For the purposes of the present report, only source angles in the azimuthal plane (at 0°/180° elevation) corresponding to those tested in the psychophysical localization experiment were analyzed.

Presented signals consisted of 11th order maximum length sequences [29] repeated without interruption 128 times from each loudspeaker. The MLS stimuli were presented at full 24-bit resolution at a rate of 97656.25 Hz (Tucker-Davis Technologies, RP2.1, TDT, Alachua, FL). A single sequence of the 11th order MLS is comprised of 2047 samples (2^11^−1) and is thus 20.96 ms in duration. The resulting acoustic waveforms were measured simultaneously in the left and right ear canals (pre-warmed to ~37°C, a feature of the test manikin) using the test manikin’s built-in ¼” microphones, amplified, and recorded to a PC using two A/D converter channels for off-line analysis. Measurements were completed in an unoccluded (“Control”) condition and again with each of the 4 HPDs (Combat Arms, ShotShields, EB15, Hybrid) in place.

#### Data processing and analysis

The impulse responses for each ear at each location were calculated by circular cross-correlation of the original 11th order MLS stimulus and the signal measured in-ear [29]. The impulse responses were then truncated to 512 (5.24 ms) points by a Hanning window beginning 700 samples (7.17 ms) after the start of the stimulus presentation, thereby approximately centering maximum deflection of the impulse response. This windowing procedure is designed to remove small-amplitude reflections from the wall opposing the active speaker (~ 2 m distance) that may be contained in the impulse responses.

HRTFs were derived by dividing the frequency response of the in-ear recording by that of the appropriate loudspeaker calibration measurement, thereby removing the loudspeaker and microphone frequency response from each in-ear measurement. The resulting function gives the acoustical gain and delay introduced by the head and the pinnae. To isolate the directional information conveyed by the HRTFs, we further computed *directional* transfer functions (DTFs). DTFs were calculated for each ear by dividing the HRTF for each spatial location by the geometrical mean of all the measured HRTFs across all measurement locations for that ear. The purpose of DTF computation is to remove “common” spectral features from the HRTFs to isolate their sound source direction-dependent components [30]; analysis based on raw HRTFs was also completed, and yielded the same pattern of results. Finally, the amplitude spectra of the DTFs were calculated using a 512-point fast Fourier transform (FFT) after the spectra were passed through a bank of band pass filters to extract information within narrow frequency bands. The filter bank consisted of 500 Butterworth filters, with the center frequencies spaced at intervals of 0.0143 octaves spanning from 0.25–32 kHz. The 3 dB bandwidth of filters was held constant across all frequencies at 0.0571 octaves, and the upper and lower slopes of the filters fell off at ~105 dB/octave.

#### HPD-related spectral distortions compared to localization performance

**Fig 9:**
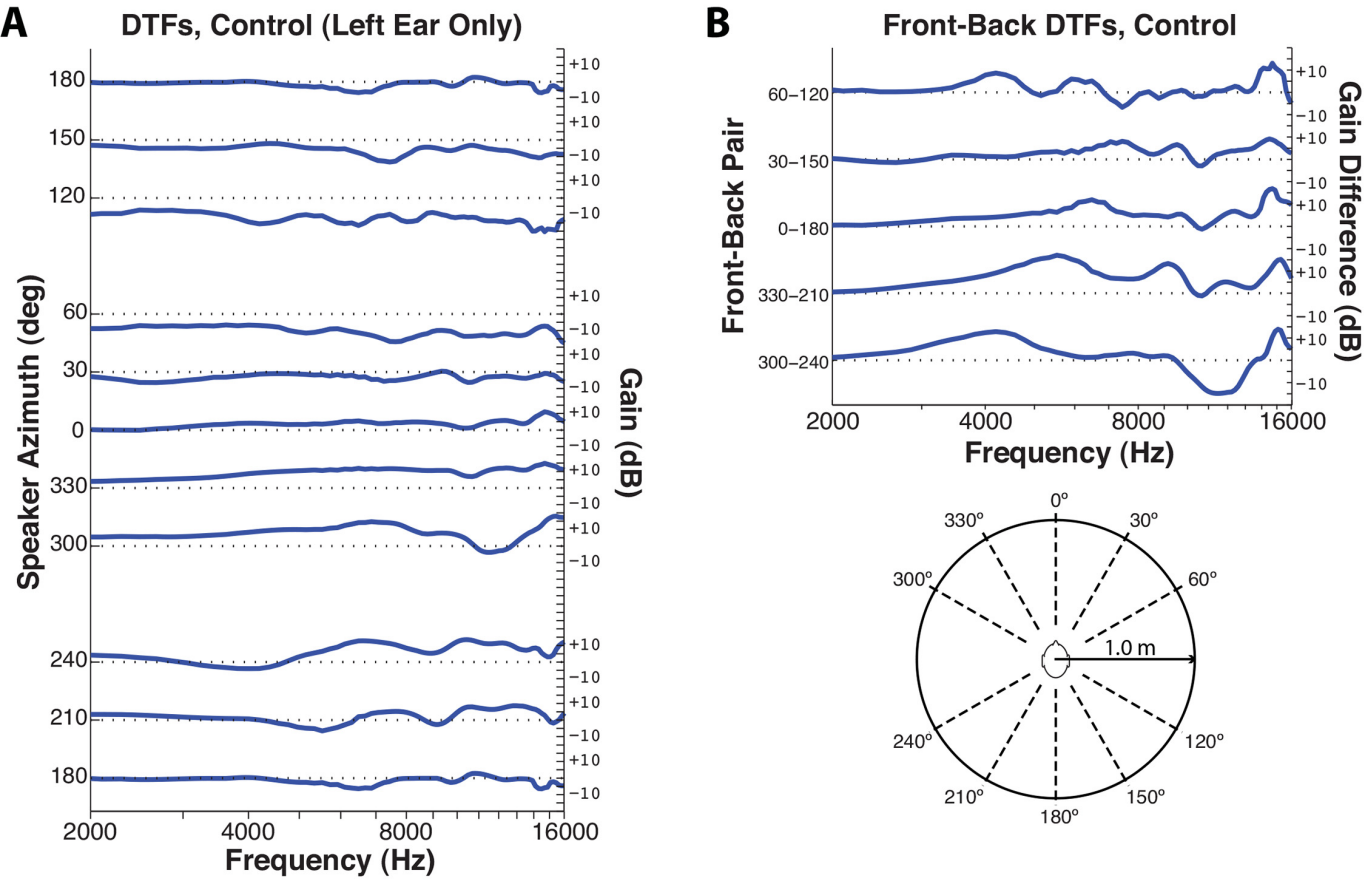
Directional transfer functions (DTFs) and differences in DTFs for front and back locations, as measured in the unoccluded (“Control” condition) left ear of an ANSI test manikin (see text). **A.** DTFs measured at the 10 locations that potentiated front-back confusions in the psychophysical experiment. Each curve gives the gain (right ordinate) unique to the specified location (left ordinate). The 180° curve is plotted twice to facilitate comparison to nearby locations. **B.** Contrasts between “front” and “back” DTFs measured in the left ear. Each curve gives the gain differences (right ordinate) across frequency that might be exploited to discriminate the specified front and back pair (left ordinate). Measurement angles are depicted in the inset graphic.

Fig 9A plots DTFs recorded at 10 source angles tested in the psychophysical localization task for the unoccluded left ear of the test manikin (symmetric data was observed in the right ear). Locations directly left (270°) and right (90°), which are logically immune to front-back confusion, are excluded. Traces for each source location are offset vertically along the y-axis (dashed gridlines). To enable better visualization of the range of frequencies over which salient DTF information is carried, data are plotted on a logarithmic scale from 2–16 kHz. Although differences across azimuth are not as clear as those observed for changes in elevation [31], DTFs differ substantially for forward and rearward source locations. Fig 9B illustrates this point clearly, giving DTF *difference* curves (simply, DTF_front_−DTF_back_) for each of the five front-back pairs present in the psychophysical testing apparatus (Fig 9B inset). For all five pairs, DTF differences approached or exceeded 10 dB in multiple regions of the spectrum.

Front-back confusions were exceedingly rare in the unoccluded condition, with 5 front-back errors total (all at 0–180) out of 3600 trials, a rate of ~0.1%. Although subjects could have gained some advantage by moving their heads during stimulus presentation [19], any such effects should have been minimal as stimuli were brief, subjects were explicitly instructed to delay any head movements until after stimulus presentation, and head position was monitored online during testing for premature movements (subjects were reinstructed when such movements were detected). Therefore, we analyzed HPD DTFs under the assumptions that (1) modifications of DTFs by HPDs were the primary source of increased front-back confusions during HPD use, (2) the left and right ears carried identical (mirror symmetric) information, and thus (3) the magnitude of change (“distortion”) in DTFs at either ear should co-vary with the incidence of psychophysical front-back errors.

**Fig 10:**
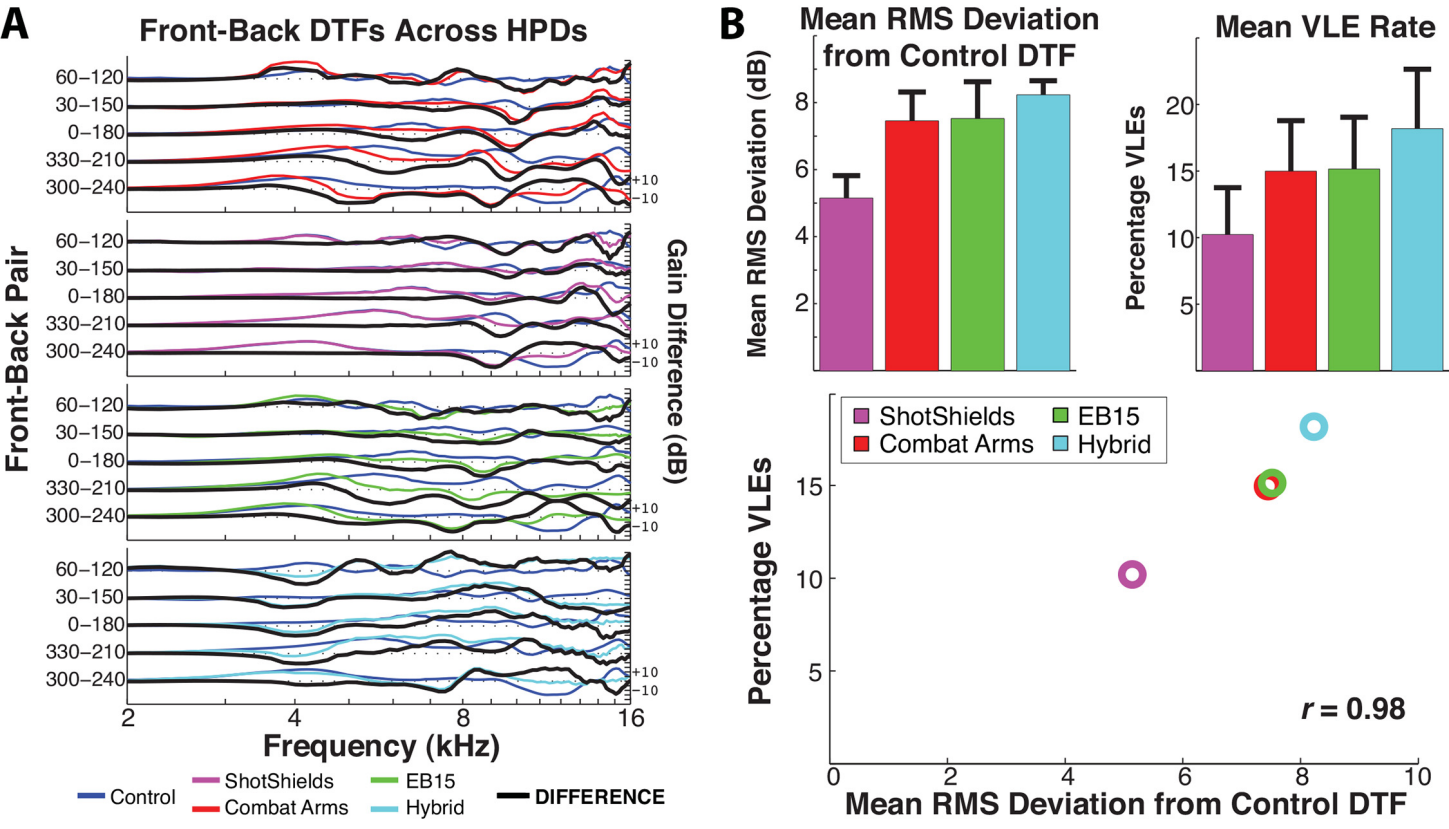
Hearing protective device (HPD)-related distortions of front-back directional transfer functions (DTFs). **A.** Front-back DTF contrasts (see Fig 9) are given for Control measurements (as in Fig 9B) and the four tested HPDs (see legend). The black curve gives the difference (in dB) between HPD and unoccluded contrasts. **B.** Means across all five potential front-back confusions are given for (upper left panel) the root-mean-square (RMS) amplitude (in dB) of black curves in (**A**) and for (upper right panel) the rate of very large errors (VLEs) in psychophysical testing, across all tested HPDs. The lower panel plots mean VLE rate against DTF distortion, suggesting a very high correlation between the two.

Each panel of Fig 10A plots DTF_front_−DTF_back_ curves for Control and HPD conditions (see legend). Most importantly, the thick black curve plots the deviation of the given HPD curve from Control (unoccluded): Thus, the greater the amplitude of the black curve in each panel, the greater the distortion of front-back spectral information by the given HPD. Curve amplitude can be summarized by its root-mean-square value (across frequencies 2–16 kHz), thus expressing the mean absolute deviation of the HPD curve from Control. Fig 10B plots these values (upper left) averaged across all five front-back pairs, effectively giving the grand mean DTF_front_−DTF_back_ distortion for each HPD. For comparison, at the upper right of Fig 10B, *psychophysical* front-back errors are summarized across devices as the grand mean percentage of trials on which subjects experienced VLEs for each device. Finally, in the lower panel of Fig 10B, mean VLE rate is plotted as a function of mean DTF distortion. While it would be most desirable to relate the performance of individual subjects to individualized acoustic measurements, the relationship between subject performance and the ANSI head measurements is striking, with a Pearson correlation coefficient of 0.98.

## Discussion

Hearing protection is important for individuals who occupy noisy environments. Currently available hearing protection devices cause degradations of auditory perceptual abilities, especially sound localization accuracy, that may be unacceptable for military personnel or others in high-risk environments [6–8] (see Introduction). Here we assessed several devices, including recently developed and previcously untested models, in a battery of auditory perceptual tests. All devices degraded performance, generally consistent with previous reports. Tone detection and speech recognition were moderately degraded, though somewhat less degraded by two active devices that better preserved audibility at high frequencies. Sound localization accuracy was severely degraded by all devices, due primarily to a marked increase in the incidence of “front-back” confusions.

In order to gain insight on the occurrence of front-back confusions during HPD use, we measured distortions of acoustic SS cues by all of the HPDs used in our localization task, and compared acoustic distortions to rates of front-back confusion (VLEs). Notably, the ShotShields device, which produced both the least acoustic distortion and the lowest mean front-back confusion rate, was also the smallest device. The ShotShields extended just beyond the entrance to the ear canal, and thus minimally occluded the concha, where high-frequency spectral cues in particular arise [16]. This observation argues strongly in favor of low-profile HPDs that minimally distort SS cues. Low-profile active HPDs analogous to in-the-canal or completely-in-the-canal hearing aids, for example, could provide the benefits of active HPDs (e.g., better audibility of low-intensity, high-frequency sounds), while minimally altering the SS cues that are important for situational awareness. Paired with auditory training and/or learning [9,32,33], HPDs with reduced in-ear profiles may offer a promising means of ameliorating the known deleterious perceptual consequences of HPD use.
